# Hearing loss and risk of depressive symptoms in older adults in the Health ABC study

**DOI:** 10.3389/fepid.2022.980476

**Published:** 2022-10-05

**Authors:** Danielle S. Powell, Joshua F. Betz, Kristine Yaffe, Stephen Kritchevsky, Elsa Strotmeyer, Eleanor M. Simonsick, Susan Rubin, Denise K. Houston, Sheila R. Pratt, Elizabeth Purchase Helzner, Katharine K. Brewster, Frank R. Lin, Alden L. Gross, Jennifer A. Deal

**Affiliations:** ^1^Department of Health Policy and Management, Johns Hopkins Bloomberg School of Public Health, Baltimore, MD, United States; ^2^Cochlear Center for Hearing and Public Health, Johns Hopkins University, Baltimore, MD, United States; ^3^Department of Biostatistics, Johns Hopkins Bloomberg School of Public Health, Baltimore, MD, United States; ^4^Department of Epidemiology and Biostatistics, University of California, San Francisco, San Francisco, CA, United States; ^5^Department of Psychiatry, Weill Institute for Neurosciences, University of California, San Francisco, San Francisco, CA, United States; ^6^Section on Gerontology and Geriatric Medicine, Department of Internal Medicine, Wake Forest School of Medicine, Winston-Salem, NC, United States; ^7^Department of Epidemiology, Center for Aging and Population Health, University of Pittsburgh, Pittsburgh, PA, United States; ^8^Intramural Research Program, National Institute of Aging, Baltimore, MD, United States; ^9^Department of Communication Sciences and Disorders, School of Health and Rehabilitation Sciences, University of Pittsburgh, Pittsburgh, PA, United States; ^10^Veterans Affairs (VA) Pittsburgh Healthcare System, Pittsburgh, PA, United States; ^11^Department of Epidemiology and Biostatistics, State University of New York (SUNY) Downstate Health Sciences University, Brooklyn, NY, United States; ^12^New York State Psychiatric Institute, New York, NY, United States; ^13^Department of Psychiatry, Columbia University Vagelos College of Physicians and Surgeons, New York, NY, United States; ^14^Department of Epidemiology, Johns Hopkins Bloomberg School of Public Health, Baltimore, MD, United States; ^15^Department of Otolaryngology, Johns Hopkins University School of Medicine, Baltimore, MD, United States

**Keywords:** hearing loss, depressive symptoms, older adult, depression, mental health, minority aging, epidemiology

## Abstract

**Objective:**

Hearing loss (HL) is highly prevalent among older adults and may lead to increased risk of depressive symptoms. In both cross-sectional and longitudinal analysis, we quantified the association between HL and depressive symptoms, incorporating the variable nature of depressive symptoms and characterizing by race and gender.

**Methods:**

Data were from the Health, Aging, and Body Composition study. Depressive symptoms were measured using the Center for Epidemiologic Study Depression Scale short form (CES-D 10), defined as CES-D 10 score ≥10 or treatment for depression. Hearing was defined *via* four-frequency pure-tone average (PTA) decibel hearing level (dB HL), categorized as normal hearing (PTA ≤25 dB HL), mild HL (PTA26-40 dB HL), and ≥moderate HL (PTA > 40 dB HL). Associations at baseline were quantified using logistic regression, incident depressive symptoms using Cox proportional hazard models, and change in depressive symptoms over time using growth mixture models and multinomial logistic regression.

**Results:**

Among 2,089 older adults (1,082 women, 793 Black; mean age 74.0 SD: 2.8), moderate or greater HL was associated with greater odds of concurrent [Odds Ratio (OR):2.45, 95% CI:1.33, 4.51] and incident depressive symptoms [Hazard Ratio (HR):1.26, 95% CI:1.00, 1.58]. Three depressive symptom trajectory patterns were identified from growth mixture models: low, moderate increasing, and borderline high depressive symptom levels. Those with moderate or greater HL were more likely to be in the borderline high depressive-symptom trajectory class than the low trajectory class [Relative Risk Ratio (RRR):1.16, 95% CI:1.01, 1.32].

**Conclusions:**

HL was associated with greater depressive symptoms. Although findings were not statistically significantly different by gender and race, estimates were generally stronger for women and Black participants. Investigation of psychosocial factors and amelioration by hearing aid use could have significant benefit for older adults' quality of life.

## Introduction

Two-thirds of adults over 70 years have a hearing loss (HL), with prevalence rising with each additional decade (1). Although traditionally viewed as primarily impacting communication, increasing evidence indicates HL may lead to clinical depression or depressive symptoms (2–4), as older adults with HL may avoid or have difficulty engaging in social situations due to problems communicating. Further, changes in brain structure and function related to HL may directly lead to increased vulnerability for depressive symptoms or result in behaviors that increase social isolation and clinical depression risk (5).

Prior studies have found HL is independently associated with depressive symptoms in older adults, yet discrepancies exist on magnitude and degree of hearing loss associated (6–10), and longitudinal studies of hearing and depressive symptoms are limited (6–8, 11). Given that depressive symptoms are commonly influenced by life events, repeated measures are essential. Recent work in the Health, Aging, and Body Composition (Health ABC) Study (9) found people with HL at baseline had greater odds of a trajectory of increasing depressive symptoms and of persistently high depressive symptoms, than people with intact hearing at baseline.

The ability to communicate effectively includes measured hearing ability, as well as social, psychosocial, and life circumstances. Therefore, aspects of one's socially defined race and gender may present different influences on risk of depressive symptoms with HL (12–14). Specifically, among women, prior work has shown women place a greater emphasis on network with friends and family and emotional intimacy than men, actions which have demonstrated protection against depressive symptoms but which may be adversely impacted by hearing loss (12). Moreover, the psychosocial stress from inclusion in a marginalized group among Black individuals has been well-documented (13, 14). This prolonged psychosocial stress has been linked to increased risk for depressive symptoms (14) and may be exacerbated further among hearing impaired Black older adults and might therefore lead to faster or poorer trajectories of symptoms over time. Hearing loss has become an established risk factor for depression. However, we do not yet know if this association differs by race or gender. While hearing loss has a higher prevalence in Whites, we hypothesize with our study that the association between hearing loss and depressive symptoms may be stronger in Blacks and emphasize the need to address disparities in hearing care. Comparison of length of depressive symptoms has been challenging in prior work due to minimal study that includes both cross-sectional and longitudinal incorporation of symptoms within the same population. Our study aims to expand upon prior work with cross-sectional and longitudinal investigation of the association between audiometrically-assessed HL and depressive symptoms, overall and by race and gender. Using data from Health ABC, we aimed to investigate the degree to which HL is associated with (i) greater cross-sectional prevalence of depressive symptoms at baseline; (ii) greater incidence of new depressive symptoms among participants without depressive symptoms at baseline over 10 years; (iii) and a poorer trajectory of depressive symptoms over time. We hypothesized HL would be associated with greater risk for the presence and new occurrence of depressive symptoms, and larger changes in severity of depressive symptoms, particularly among women and Black participants.

## Materials and methods

### Study population

Health ABC is a prospective study of 3,075 well-functioning (no difficulty walking ¼ mile or climbing 10 steps), community-dwelling Black and White men and women aged 70–79 years, initiated in 1997–1998 in Pittsburgh, Pennsylvania and Memphis, Tennessee (15). Follow-up consisted of annual or bi-annual clinical examinations and 6-month interim phone calls, terminating in 2016. This study was approved by the Institutional Review Boards of all participating institutions.

Participants were excluded from this analysis if they: (1) did not attend year 5 when audiometry was performed (*n* = 779); (2) had incomplete audiometric data (*n* = 93); (3) were missing covariates (*n* = 99); or (4) were missing baseline depressive symptom scores or reported a history of treatment for depression (*n* = 15), for an analytic sample of 2,089. Analyses of the rate of longitudinal change of depressive symptoms further excluded those with fewer than two completed measures of depressive symptoms (*n* = 1). For the longitudinal investigation of incident clinical depression or depressive symptoms, those with high depressive symptoms at baseline (*n* = 111) were excluded.

### Measures

#### Depressive symptoms

**Figure 1:**
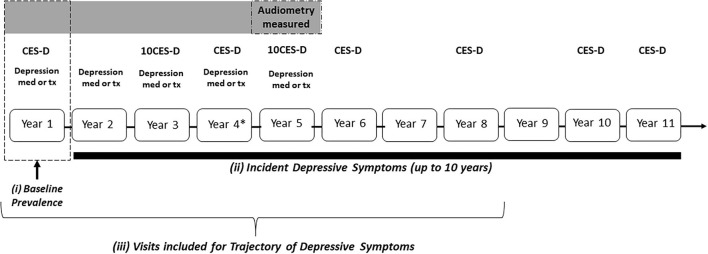
Study design of the health, aging, and body composition study. Depression med, reported use of antidepressant; tx, reported clinical treatment for depression. *CES-D measured at Year 4 not included for trajectory measures.

A comprehensive assessment of clinical depression and depressive symptoms was obtained using multiple measures (Figure 1). At baseline, a history of self-reported treatment for clinical depression or history of antidepressant use intended for depression was assessed. The standard 20-item Center for Epidemiological Studies Depression Scale [(CES-D); years 1, 4, 6, 8, 10, and 11] or the abbreviated 10-item [(CES-D 10); years 3 and 5] was administered to participants, allowing for up to 8 measurements. Medication inventories, including dosages and indication of all medications and supplements, as well as self-reported treatment for clinical depression were collected during years 2, 3, 5, and 6. Treatment for depression was defined as use of antidepressant medication with an indication of depression or self-reported treatment for clinical depression. To allow for maximum comparability across years, scores on the 20-item CESD were converted to the 10-item scale (16, 17). Scores ≥ 10 on the CES-D 10 have been shown to be highly sensitive in identifying participants with clinically meaningful depressive symptoms (16, 17). At baseline, prevalent depressive symptoms were defined as CES-D 10 score ≥ 10 or self-reported current treatment for clinical depression. Incident depressive symptoms was defined as a change in CES-D 10 scores to a score ≥ 10 from a score of <10 or reported incident treatment for depression between years 2-11. Baseline for incident depressive symptoms was defined as year 1 (study baseline).

#### Hearing measures

Audiometry was performed at year 5 (2001–2002). Air-conduction thresholds were obtained for each ear at 500, 1,000, 2,000, 4,000, and 8,000 Hz in a sound-proof booth using an audiometer (Maico MA40) and supra-aural earphones (TDH 39). A speech-frequency pure tone average (PTA) of hearing thresholds obtained at 500–4,000 Hz was calculated for the better hearing ear. Hearing level was evaluated continuously in decibels hearing level (dB HL) and categorized based on clinical cutpoints (no HL: ≤25 dB HL; mild impairment: 26–40 dB HL; moderate or greater impairment >40 dB HL). In secondary analysis, we investigated if hearing aid use, ascertained *via* self-report at years 1 and 5 (binary yes/no), attenuated the influence of HL on prevalent or incident depressive symptoms.

#### Other covariates

Demographic information collected at baseline (year 1) included age, gender (men vs. women defined at birth), race (Black vs. White), education (less than high school, high school, or post-secondary education), marital status (never married, married, widowed/divorced/separated), living alone, and study site. Health-related factors were treated as time-fixed at baseline, including smoking (never, former, current), hypertension (systolic blood pressure ≥140 mm Hg and/or diastolic blood pressure >90 mmHg) history of reported cardiovascular disease; reported history of stroke, and type 2 diabetes mellitus (use of diabetes medications and/or fasting glucose ≥120 mg/dL).

### Statistical analyses

Baseline characteristics by hearing were compared using the Wilcoxon rank-sum and Fisher's exact tests.

#### Prevalence of depressive symptoms at baseline

Differences in the prevalence of baseline depressive symptoms and current treatment for clinical depression by category of HL were examined using logistic regression. Models were adjusted for demographic and health factors. All analyses were further stratified by race and gender to investigate potential risk modification. As a previous history of depressive episode is known to increase risk for future episodes, in sensitivity analyses we adjusted for history of reported depression prior to baseline and hearing aid use. Those participants who self-reported antidepressant medication use at baseline but no history of depressive symptoms or current treatment for depression were not included in the analysis as these individual's medication use may influence responses to depressive symptom questionnaires.

#### Incident significant depressive symptoms or treatment for clinical depression

The association between HL and incident significant depressive symptoms was modeled using discrete time proportional hazard models among those with CES-D 10 scores <10 at baseline with no reported treatment for depression. Proportionality was assessed using Schoenfeld residuals (18). Year 1 was used as the time origin. Individuals had an event once they reported initiation of treatment or medication intended for depression, or first CES-D 10 score ≥ 10. Participants were censored at the first missing observation. Models were adjusted for age, education, gender, and race due to limited number of events. In secondary analyses, models were stratified by race and gender to investigate potential modification of risk. Sensitivity analyses further adjusted for hearing aid use at Year 5 when hearing was measured and at baseline in separate analysis, and history of treatment for clinical depression at baseline.

#### Trajectory of depressive symptoms over time

Depressive symptoms can vary widely within individuals over time, making it difficult to distinguish or identify trajectories *a priori*. As one goal of this analysis was to identify groups of individuals with overall similar depressive symptom trajectories, we characterized change in depressive symptoms over time using a group-based trajectory modeling approach. Trajectories of depressive symptoms were evaluated over the first 10 years of follow-up using growth mixture models (GMM), a form of trajectory modeling that models individual variation in identified developmental paths over time as distinct classes defined by levels and trajectories of CES-D 10 scores and does not require investigator defined *a priori* definitions of trajectories. GMM uses a parametric finite mixture model and accommodates individual heterogeneity within each identified subgroup (19–21). Mean trajectories of CES-D 10 scores were estimated in the full analytic sample of 2,089 adults using MPlus 8.0 (22) (years 1, 3, 5, 6, 8; Year 4 was not included as medication use was not collected for the year but collected for the other years). Years of CES-D 10 were selected to include trajectory years with the richest depressive symptom data for analysis and include measures around when audiometric testing was performed. We used a maximum likelihood estimator and freely estimated within class variances. We determined the number of trajectory classes based on recommended procedures (21), using measures of AIC, BIC, Bootstrapped likelihood ratio test, and the Lo, Mendell, Rubin likelihood ratio test. Based on these procedures, the best fitting model included 3 trajectories of depressive symptoms for our subsequent analyses: low trajectory pattern, moderate increasing trajectory pattern, and a borderline high trajectory pattern.

To investigate if HL is associated with latent class membership of depressive symptom trajectories, we used multinomial logistic regression, estimating the relative risk ratio of depressive symptom trajectory by hearing status from baseline to year 11 (normal hearing as reference). Models were adjusted for demographics and health factors and antidepressant medication use as the use of antidepressant medications may influence performance on the CES-D 10. Those participants who self-reported antidepressant medication use but no history of depressive symptoms or current treatment for depression were not included in the analysis (*n* = 14). All analyses were again stratified by race and gender.

Significance testing for all analyses was conducted using 2-sided tests (type I error rate = 0.05). Logistic, discrete time proportional hazard, and multinomial logistic regression models were performed using STATA version 15 (StataCorp, 2015. Stata Statistical Software: Release 14. College Station, TX: StataCorp LP).

## Results

### Demographic and clinical characteristics

Of the 2,089 participants included in our prevalence analysis at baseline, 861 (41.2%) had normal hearing, 793 (38.0%) had a mild HL, and 435 (20.8%) had a moderate or greater HL, 182 (8.7%) reported a history of depression at baseline, 79 (8.3%) had clinically significant depressive symptoms or indicated current treatment for clinical depression at baseline, and 89 (4.5%) had incident significant depressive symptoms (11 participants noting both incident treatment for depression and scoring ≥10 on the CESD10). A total of 189 participants (8.7%) reported a history of depressive symptoms at study baseline. The median CES-D 10 score at baseline was 2.0 (IQR: 0.0–4.0) (Table 1). Those with normal hearing were more likely to be younger, women, Black and have a higher education level than those with HL. Just under 9% (186) reported ever using a hearing aid at baseline.

**Table 1:** Baseline characteristics of the analytic sample and by hearing status in Health ABC (*N* = 2,089).

	**Overall (*N* = 2,089)**	**Normal hearing (*N* = 861)**	**Mild hearing loss (*N* = 793)**	**Moderate or greater hearing loss (*N* = 435)**	***p*-value^a^**
	***N* (%)**	***N* (%)**	***N* (%)**	***N* (%)**	
Baseline age, mean (SD)	74.0 (2.8)	73.3 (2.7)	74.2 (2.8)	74.8 (2.9)	<0.001
PTA, mean (SD)	30.3 (13.5)	18.2 (5.1)	32.6 (4.3)	50.4 (9.1)	<0.001
Black	793 (38.0)	406 (47.2)	275 (34.7)	112 (25.7)	<0.001
Women	1,082 (51.8)	531 (61.7)	398 (50.2)	153 (35.2)	<0.001
Education					0.033
Post-secondary	933 (44.7)	391 (45.4)	364 (45.9)	178 (40.9)	
High school grade	693 (33.2)	288 (33.4)	269 (33.9)	136 (31.3)	
Less than high school	463 (22.2)	182 (21.1)	160 (20.2)	121 (27.8)	
Memphis study center	1,023 (49.0)	392 (45.5)	395 (49.8)	236 (54.3)	0.010
Marital status					0.012
Never married	102 (4.9)	41 (4.8)	40 (5.0)	21 (4.8)	
Married	1,219 (58.4)	466 (54.1)	479 (60.4)	274 (63.0)	
Widowed, divorced, separated	768 (36.8)	354 (41.1)	274 (34.6)	140 (32.2)	
Live alone	603 (28.9)	268 (31.1)	218 (27.5)	117 (26.9)	0.16
Baseline diabetes	727 (34.8)	279 (32.4)	282 (35.6)	166 (38.2)	0.10
BMI, mean (SD)	27.4 (4.7)	27.4 (4.9)	27.4 (4.8)	27.1 (4.1)	0.48
Smoking history					<0.001
Never smoker	950 (45.5)	434 (50.4)	355 (44.8)	161 (37.0)	
Former smoker	969 (46.4)	353 (41.0)	384 (48.4)	232 (53.3)	
Current smoker	170 (8.1)	74 (8.6)	54 (6.8)	42 (9.7)	
Baseline history of CVD	1,379 (66.0)	575 (66.8)	523 (66.0)	281 (64.6)	0.73
Baseline history of depression	182 (8.7)	69 (8.0)	68 (8.6)	45 (10.3)	0.37
CES-D 10 score, median (IQR)	2.0 (0.0–4.0)	2.0 (0.0–4.0)	2.0 (0.0–4.0)	2.0 (0.0–4.0)	0.52

PTA, pure tone average; SD, standard deviation; BMI, body mass index; CVD, cardiovascular disease; CES-D 10, Center for Epidemiologic Studies Depression scale short form; Mild hearing loss = PTA 25-40 dB HL; Moderate or greater hearing loss = PTA > 40 dB HL.

^a^Baseline characteristics were compared using Wilcoxon rank-sum tests and Fisher's exact tests.

*p < 0.05.

### Prevalence of depressive symptoms at baseline

Overall, compared to normal hearing, those with a moderate or greater HL experienced 2.45 times the odds [95% confidence interval (CI): 1.33, 4.51] of clinically significant depressive symptoms (Table 2). Although *p*-values for interaction were not significant (*p* = 0.176 for gender and *p* = 0.782 for race), this association was stronger in magnitude and only statistically significant for women and Black participants.

**Table 2:** Cross-sectional prevalence of clinically significant depressive symptoms or treatment for depression at baseline (1997–1998) by hearing category, race, and sex in Health ABC (*N* = 2,089).

	**Normal hearing**	**Mild hearing loss**	**Moderate or greater**	*P* for trend^*^
			**hearing loss**	
	**Odds ratio [95% CI]**	**Odds ratio [95% CI]**	**Odds ratio [95% CI]**	
Overall (*N* = 2,089)	1.0 (Reference)	1.38 [0.78, 2.43]	**2.45 [1.33, 4.51]**	0.004
Women (*N* = 1,082)	1.0 (Reference)	1.70 [0.83, 3.47]	**2.89 [1.30, 6.43]**	0.009
Men (*N* = 1,007)	1.0 (Reference)	0.92 [0.35, 2.44]	1.81 [0.69, 4.72]	0.190
Black (*N* = 793)	1.0 (Reference)	2.24 [0.93, 5.42]	**3.35 [1.25, 9.01]**	0.013
White (*N* = 1,296)	1.0 (Reference)	0.96 [0.45, 2.06]	1.84 [0.85, 3.96]	0.126

Normal hearing defined as PTA <25 dB HL, Mild hearing loss PTA > 25 and ≤40 dB HL; Moderate or greater hearing loss as PTA > 40 dB HL.

Models adjusted for demographic (age, gender, race, education) and health factors (study site, marital status, living alone, diabetes, Body mass index, cardiovascular disease history).

*α = 0.05. Bold values refers to statistically significant *p* < 0.05.

Among women, compared to participants with normal hearing, those with moderate or greater HL had 2.89 times the odds of clinically meaningful depressive symptoms (95% CI: 1.30, 6.43). Black participants with moderate or greater HL or mild HL demonstrated over 3 times (OR: 3.35, 95% CI 1.25, 9.01) greater odds of depressive symptoms compared to Black participants with normal hearing.

Results were similar in sensitivity analyses adjusting for reported history of treatment for depression at baseline and antidepressant use without reported treatment for depression. Exploration of hearing aid use demonstrated no overall association with prevalent depressive symptoms at baseline.

### Incidence of significant depressive symptoms by hearing status

Hearing loss was associated with a greater risk for incident depressive symptoms (CES-D 10 ≥ 10) for those with a moderate or greater HL compared to normal hearing (HR: 1.26, 95% CI: 1.00, 1.58) after accounting for demographic and health factors (Table 3). We did not find significant differences in the association between hearing status and incident depressive symptoms by race or gender.

**Table 3:** Incidence of new clinically significant depressive symptoms or incident treatment for depression over 10 years (1997/1998–2007/2008) by hearing category, race, and sex in Health ABC (*N* = 1,978).

	**Normal hearing**	**Mild hearing loss**	**Moderate or greater**	*P* for trend^*^
			**hearing loss**	
	**HR (95% CI)**	**HR (95% CI)**	**HR (95% CI)**	
Overall (1,978)^a^	1.0 (Reference)	1.15 [0.96, 1.39]	**1.26 [1.00, 1.58]**	0.037
Women (1,014)^b^	1.0 (Reference)	1.15 [0.91, 1.46]	1.33 [0.97, 1.83]	0.062
Men (964)^b^	1.0 (Reference)	1.16 [0.86, 1.55]	1.20 [0.86, 1.66]	0.273
Black (744)^b^	1.0 (Reference)	1.11 [0.85, 1.45]	1.22 [0.86, 1.74]	0.239
White (1,234)^b^	1.0 (Reference)	1.20 [0.90, 1.48]	1.26 [0.94, 1.68]	0.066

Normal hearing defined as PTA <25 dB HL, Mild hearing loss PTA > 25 and ≤40 dB HL; Moderate or greater hearing loss as PTA > 40 dB HL.

^a^Overall model fully adjusted for demographics (age, gender, race, education).

^b^Stratified models adjusted for demographics (age, gender/race, education) and excluding those reporting antidepressant use but not treatment for depression.

*α = 0.05. Bold values refers to statistically significant *p* < 0.05.

Results remained consistent after adjustment for history of treatment for depression at baseline. Self-reported hearing aid use at baseline or at Year 5 demonstrated no association with incident depressive symptoms.

### Trajectory of depressive symptoms over time by hearing status

**Figure 2:**
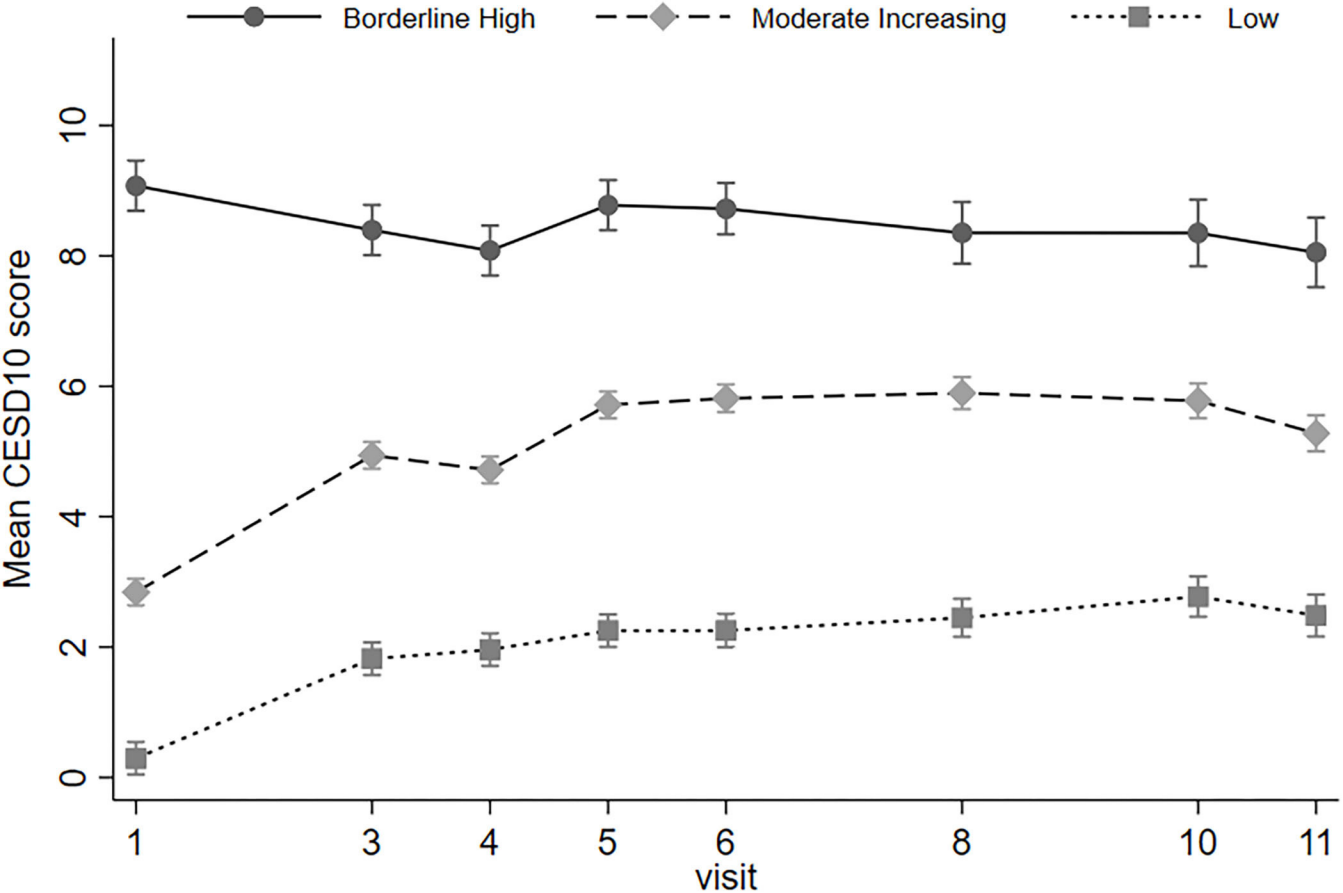
Mean CES-D 10 score by depressive symptom trajectory across 10 years of follow-up (1997/1998–2007/2008) in Health ABC study. Depressive symptom trajectory created using Center for Epidemiologic Study Depression scale short-form (CES-D 10) responses from visits 1, 3, 5, 6, and 8. 27.1% of participants were in the moderate increasing trajectory; 66.6% in the low depressive symptom trajectory; 6.3% in the borderline high depressive symptom trajectory.

We identified three depressive symptom trajectory patterns: low, moderate increasing, and borderline high depressive symptom levels (Figure 2). Among the 2,088 participants included in the baseline analytic cohort, 1,390 (66.6%) had low levels of depressive symptoms, 566 (27.1%) had moderate increasing symptoms, and 132 (6.3%) had borderline high symptom levels. The mean posterior probabilities for group membership for each trajectory are 0.967 for low, 0.832 for moderate increasing, and 0.866 for borderline high, suggesting strong classification quality.

Overall, for participants with moderate or greater hearing loss, the relative risk ratio of a borderline high depressive symptom trajectory compared to low trajectory was 1.16 (95% CI: 1.01, 1.32), but hearing loss was not associated with increased risk for membership in the moderate increasing depressive symptoms vs. low depressive symptoms group (RRR = 0.96, 95% CI: 0.87, 1.05) (Table 4).

**Table 4:** Relative risk ratio of depressive symptom trajectory over 10 years (1997/1998–2007/2008) by hearing category, race, and sex in Health ABC (*N* = 2,088).

	**Overall**	**Women**	**Men**	**Black**	**White**
**Borderline high trajectory (*****N*** **=** **132)**
Mild hearing loss	1.06	1.10	1.03	**1.24**	0.95
	[0.95, 1.18]	[0.95, 1.26]	[0.86, 1.23]	**[1.04, 1.48]**	[0.82, 1.09]
≥Moderate hearing loss	**1.16**	**1.25**	1.09	**1.58**	1.05
	**[1.01, 1.32]**	**[1.03, 1.52]**	[0.89, 1.32]	**[1.24, 2.03]**	[0.89, 1.24]
**Moderate increasing trajectory (*****N*** **=** **566)**
Mild hearing loss	**0.91**	0.93	0.89	1.07	**0.83**
	**[0.84, 0.98]**	[0.84, 1.03]	[0.80, 1.00]	[0.94, 1.21]	**[0.75, 0.91]**
≥Moderate hearing loss	0.96	1.01	0.94	**1.43**	**0.83**
	[0.87, 1.05]	[0.86, 1.17]	[0.83, 1.07]	**[1.19, 1.73]**	**[0.74, 0.93]**
**Low trajectory (*****N*** **=** **1,390)**
Mild hearing loss	Reference	Reference	Reference	Reference	Reference
	–	–	–	–	–
≥Moderate hearing loss	Reference	Reference	Reference	Reference	Reference
	–	–	–	–	–

Depressive symptom trajectory created using Center for Epidemiologic Study Depression scale short-form (CES-D 10) responses from visits 1, 3, 5, 6, and 8.

Models adjusted for demographics (age, gender, race, education) and health factors (study site, marital status, living alone, diabetes, Body mass index, cardiovascular disease history, antidepressant medication use).

27.1% of participants were in the moderate increasing trajectory; 66.6% in the low depressive symptom trajectory; 6.3% in the borderline high depressive symptom trajectory.

Normal hearing defined as PTA <25 dB HL, Mild hearing loss PTA > 25 and ≤40 dB HL; Moderate or greater hearing loss as PTA > 40 dB HL. Bold values refers to statistically significant *p* < 0.05.

However, in stratified analyses, Black participants with moderate or greater hearing loss had a greater risk of being in the borderline high (RRR = 1.58, 95% CI: 1.24, 2.03) and moderate increasing trajectory groups (RRR = 1.43, 95% CI: 1.19, 1.73), compared to low depressive symptoms over time, By contrast, for White participants, hearing loss was associated with a lower risk of inclusion in the moderate increasing compared to low trajectory (RRR = 0.83, 95% CI: 0.74, 0.93); *P*-value for Wald test for interaction by race = 0.002.

In gender-stratified analyses, *P*-values for interaction were not significant but estimates of the association of hearing loss and trajectory group membership were null or inverse for men. Women with moderate or greater HL had a 1.25 (95% CI: 1.03, 1.52) greater relative risk of borderline high trajectory vs. low trajectory.

## Discussion

In this demographically diverse population-based cohort of 2,089 older adults in the U.S. with up to 10 years of follow-up, we investigated the impact of HL on depressive symptoms using a comprehensive definition of depression incorporating self-reported history of depression, medication use, and a depressive symptom questionnaire. Those with moderate or greater HL (vs. normal hearing) demonstrated 2.5-fold greater odds of clinically significant depressive symptoms at baseline, and a ~25% increase in the risk of incident clinically significant depressive symptoms over time. Analyses of depressive symptoms over time suggested that moderate or greater hearing loss was associated with persistently higher depressive symptoms over time.

Our results are consistent with the preceding work by Brewster et al. (9), which demonstrated an association between low and mid-frequency hearing status and depressive symptoms based on CES-D 10 scores at year 5 of the Health ABC study. Their analysis found 1.63 greater odds of an increasing depressive symptom trajectory and 1.85 greater odds of a consistently high depressive symptom trajectory for those reporting any age-related HL compared to those reporting no HL. This prior work uniquely accounted for the complex nature of late-life depression by capitalizing on the longitudinal depression measures in Health ABC and depressive symptom trajectory over time. With expansion on this prior work we reached similar findings, using a multi-faceted characterization of depression symptoms and conducting stratified analyses to investigate potential differences by race and gender. While consistent with these previous findings, our analysis undertook a more comprehensive approach within the same study sample to characterize the association in both cross-sectional and longitudinal models using an established and validated depression questionnaire and the gold standard of hearing assessment. We found that severity of depressive symptoms at the start of follow-up was strongly associated with depressive symptoms measured over time; our classification of depressive symptom trajectory was dependent upon the symptom severity at baseline.

Other studies have also reported associations between hearing loss and depressive symptoms. Cacciatore et al. (23) suggested a positive relationship (*r* = 0.85) between older adults with hearing difficulty and depressive symptom scores. A meta-analysis (24) including studies of chronic diseases and risk for depression in old age reported a pooled odds ratio of 1.71 for prevalent depression among those with poor hearing and a pooled relative risk of 1.92 for incident depression among those with poor hearing compared to normal. An additional meta-analysis (6) of the hearing-depression association supports an overall greater odds of depression for those with hearing loss with significant heterogeneity in results, yet the authors comment on many limitations of the included study including mixed study design and measurements used. Longer follow-up for depressive symptoms starting in late mid-life may shed light on sensitive periods for development of depressive symptoms among hearing impaired older adults or unique characteristics of those with stable trajectories.

We hypothesized that we would observe differences in the association between hearing impairment and depressive symptoms by race and gender, which could highlight the interconnectedness between influences of social structure, communication ability, and depressive symptoms for older adults. Prior work has identified differences in late-life depression by gender and race (14, 25–28) yet few, if any, prior studies have attempted to quantify these differences within the hearing and late-life depression association. Though overall support of an interaction between HL and race or gender was generally not found, we did observe differences in magnitude of association, with stronger estimated associations for women and Black participants, particularly for reporting higher depressive symptoms at baseline, and persistent depressive symptoms over time.

Our use of several methods to represent the phenomenology of depressive symptoms helps to better capture the complex presentation of late-life depression. Major Depressive Disorder is generally less prevalent in older compared to younger adults (29), but it is possible that even one episode of depression is associated with significant negative outcomes (i.e., dementia, increased mortality, slower medical recoveries, increased disability) (29, 30). Late-life depression often has a differing presentation than at younger ages, which warrants greater understanding for potential intervention (31, 32). Older adults are more likely to endorse loss of interest in life or activities or express somatic symptoms (29). Although much remains in the identification of risk factors for late-life depression, a complex relationship between biological changes and vulnerabilities, stressful events, curtailment of daily activities, and self-criticism may predispose an older adult to depressive symptoms (5, 28, 29). HL may therefore increase risk for late-life depression because of withdrawing from social activities or reduced engagement from difficulty communicating. Hearing impaired older adults also may become discouraged by inability to have quality connections with others leading to downstream psychosocial effects like social isolation, loneliness, and depression. Additionally, the increased cognitive processing required to compensate for hearing loss may lead to executive dysfunction (33)—itself a risk factor for late-life depression (34). A biological basis and brain changes associated with HL has been hypothesized as a mechanism for the association, including deafferentiation-induced atrophy of the auditory cortex and frontotemporal regions as well as abnormal cognitive control and emotion processing networks (35, 36), each warranting further study (5).

Even with the modest increased risk demonstrated in our analysis, consideration of HL as a risk factor for clinical depression is notable owing to the high prevalence of HL and potential treatability with hearing aids and other types of hearing technologies. Further characterization of how the intersectionality of race and gender (i.e., Black women, White women, Black men, and White men) among hearing impaired older adults may influence depressive symptoms presents opportunity for innovation in how each group may cope with HL given life and social circumstances. Management of HL has the potential to improve existing intervention strategies for late-life depression. The ability to maintain quality communication with physicians and adequately engage in intervention strategies like cognitive behavioral therapy for late-life depression deserves further study, has the potential to supplement current management strategies. This may be especially important as we consider ways to improve access and ways to communicate to evidence-based treatments for depression among people of color. Reducing the clinical and social burden of late-life depression through aural rehabilitation or combined existing depression intervention strategies could have far-reaching incidental benefit.

Prior research on the effect of race and gender on late-life depression has indicated increased risk among women compared to men and Black individuals compared to non-Hispanic White individuals. When investigating racial differences, a cross-sectional study of over 50,000 older adults (27) indicated significantly greater odds of anhedonia (i.e., inability to feel pleasure), psychomotor symptoms, and sadness among Black participants compared to non-Hispanic White participants. Results were robust even after adjustment for social and health determinants such as socioeconomic status, lifestyle factors or comorbidities. Moreover, it is possible HL increases vulnerability to the social and emotional strain and inequalities faced by many minorities in the U.S. (14). This vulnerability might therefore predispose minority individuals to increased depressive symptoms. Further, it is possible the prolonged communication strain from significant HL may add to that from societal inequalities and racial disparities, leading to the higher risk in our analysis over time for Black participants. Known disparities in access to health care, utilization of health services, and delays to the initiation of psychosocial treatment by race exist (27), which may potentially exacerbate this vulnerability. Further characterization of how HL may have differing social and emotional effects by race may highlight avenues for intervention of depression.

Prior results also suggest greater odds of depressive symptoms including depressed mood and somatic complaints among older women compared to older men (28). Our findings remained consistent across measures of hearing difficulty. Prior hypotheses have suggested social factors may lead older women to place greater emphasis than older men on spoken communication to maintain connection (5, 37). Therefore, due to these social influences, HL may have a greater impact on feelings of connection in women and result in the higher baseline prevalence and greater dose response observed in our study. Future investigation of the inter-relational effect of race and gender among hearing impaired older adults may aid in determining groups at the greatest risk of depressive symptoms as well as provide opportunity for personalized intervention for late-life depression.

Although the longitudinal nature of our study is a strength, we acknowledge the limitation of audiologic measures completed 4 years after baseline. We elected to use the full 9 years of rich depressive symptom/depression measures available by using Year 1 as baseline but it presented a chronological gap from when hearing was measured. However, for most older adults, hearing changes very gradually at a rate of 1–2 dB per year (38) and is an approach which has been used in other studies (39, 40). For example, to consider our sample with hearing loss since the exposure of interest, the mean PTA of hearing aid users at study baseline was 54 dB HL (SD 8.4) compared to 53 dB HL (SD: 9.1) at Year 5. Therefore, the time between baseline and when audiometry was performed likely only presented a minimal change in hearing. We therefore did not expect significant misclassification by hearing category—any misclassification would likely have led to a conservative estimate of the association observed between hearing and depressive symptoms. Furthermore, the measures collected for depressive symptoms at Years 1–4 are significantly stronger and more comprehensive than at Year 5 and later when hearing was measured due to inclusion of medication information and more frequent depressive screening completion, which strengthened our incident and longitudinal models and guided our selection of using Year 1 as baseline for all analysis. When we completed our longitudinal analysis which utilizes the most data using Year 5 as baseline, our results continued to suggest greater association with poorer depressive symptoms over time for those with a moderate or greater hearing loss. Due to the limited number of incident depressive symptoms in our study sample, our incidence analysis was limited in the number of covariates adjusted for and only adjusted for demographics. We cannot rule out that other factors may influence our study findings, however our analysis lays a foundational guidance for future investigation. We were additionally not able to assess medication use for treated depression beyond year 6. Albeit the CES-D 10 demonstrates good sensitivity in identifying those with significant depressive symptoms, it is not a diagnostic measure for depression. It is possible this assessment may incorrectly capture constructs of depressive symptoms appropriate for older adults. We may underestimate depressive symptoms among older adults or subgroups from participants' hesitation to report depressive symptoms or seek treatment due to stigma. However, a strength of our analyses is the multiple modes by which we can assess depressive symptoms that may better capture the presence of symptoms. Due to our inclusion of both participants who both indicate treatment for depression and indication of depressive symptoms *via* questionnaire within the same definition of incident depressive symptoms, we are not able to evaluate differences in association with hearing loss by these groups or isolate those who are treated for depressive symptoms from those who indicate symptoms *via* the CESD10. While this presents a limitation to our study for some clinical purposes, our study continues to inform how presentation of depressive symptoms might inform low-risk and novel avenues for depression management. Cross-sectional measures of symptoms may be subject to episodic depressive symptoms related to situational circumstances such as stressful life events (i.e., death of a spouse) and therefore may not correctly reflect longer-term depressive symptom levels. Our study includes both cross-sectional measures, for comparability to previous studies, as well as two different means to assess longitudinal symptoms over time. This comprehensive assessment may more accurately reflect depressive symptoms in older adults through utilization of depression questionnaires, self-report history for treatment or diagnosis, and use of medications prescribed for depression.

Our results investigating reduced risk of depressive symptoms among hearing aid users yielded findings that were not statistically significant when investigating hearing aid use as measured at baseline or at Year 5, although over 90% of hearing aid users at baseline were also using a device at Year 5. However, our measure of hearing aid use included self-report hearing status. Individuals commonly over-estimate their use of a hearing aid (40) and may therefore lead to misclassification of hearing aid use and contribute to our null findings. The potential benefit of hearing aid use for management of HL and reducing depression risk is substantial. Therefore, future study with more sophisticated evaluation of hearing aid use and appropriate device fitting could have far reaching clinical and public health benefit.

In a longitudinal cohort study of older adults, results support an association between greater degree of HL and prevalent, persistent and incident clinically significant depressive symptoms, particularly for those with a moderate or greater HL. Clinical providers working with older adults might consider the patient's hearing status when addressing risk factors for late-life depression, as well as disparities in psychosocial and hearing care by race and gender. While our study did not find hearing aid use was associated with reduced depressive symptoms, further study understanding whether biological or social components of hearing loss are associated with depressive symptoms and exploration of additional management strategies for hearing loss may highlight other intervention paths to improve psychosocial outcomes for older adults.

## Data availability statement

Publicly available datasets were analyzed in this study. This data can be found at: Health, Aging, and Body Composition (HABC) Study https://healthabc.nia.nih.gov/.

## Ethics statement

Ethical review and approval was not required for the study on human participants in accordance with the local legislation and institutional requirements. Written informed consent from the participants was not required to participate in this study in accordance with the national legislation and the institutional requirements.

## Author contributions

DP, JB, JD, and AG: conceptualization. DP and JB: statistical analysis. DP, AG, and JD: manuscript drafting. JB, KY, SK, ESt, ESi, SR, DH, SP, EP, KB, and FL: manuscript revision. AG and JD: supervision. All authors contributed to the article and approved the submitted version.

## Funding

This work was supported by National Institute on Aging (NIA) contracts #N01-AG-6-2101; N01-AG-6-2103; N01-AG-6-2106; NIA grant R01-AG028050; and NINR grant R01-NR012459. DP was supported by fellowship funding through the Cochlear Center for Hearing and Public Health and by NIH/NIA T32AG000247. JD was supported by NIH/NIA grant K01AG054693. AG was supported by NIH/NIA grant K01AG050699.

## Conflict of interest

JB reports entitlement to future royalties and equity in miDiagnostics. FL reports being a consultant to Frequency Therapeutics, speaker honoraria from Caption Call, and being the director of a public health research center funded in part by a philanthropic gift from Cochlear Ltd to the Johns Hopkins Bloomberg School of Public Health.

The remaining authors declare that the research was conducted in the absence of any commercial or financial relationships that could be construed as a potential conflict of interest.

## Publisher's note

All claims expressed in this article are solely those of the authors and do not necessarily represent those of their affiliated organizations, or those of the publisher, the editors and the reviewers. Any product that may be evaluated in this article, or claim that may be made by its manufacturer, is not guaranteed or endorsed by the publisher.
